# Climate and Health

**Published:** 1895-11

**Authors:** 


Climate and Health. Edited under the direction of Prof. Willis L. Moore, Chief of Weather Bureau, by W. F. R. Phillips, M. D. Number One. A Summary of Statistics for the four weeks ended July 27th, 1895. Washington, Weather Bureau, July, 1895. Vol. I, No. 1.

This publication is a monthly periodical issued by the Weather Bureau, through suggestions made by the Secretary of Agriculture. In addition to meteriological data it contains important vital statistics.

"The statistics of mortality and morbidity are furnished by special reports of public health officials and of physicians made directly to the Weather Bureau. * * * The effort will be persistently made to secure, as nearly as practicable, accurate and trustworthy statistics concerning the sanitary conditions prevailing from week to week."

"If the discovery should be made that deaths from any given cause may be expected to occur more frequently during the manifestation of certain atmospheric phenomena, it would probably be of considerable value to the physician in the treatment of the disease, but it is doubtful if this sort of knowledge could be of service in the prevention of the disease. On the other hand, if the prevalence of a given atmospheric condition should be found to be coincident with the greater or less frequency of a certain disease, the knowledge will be useful to both the physician and the general public in indicating an insistence upon the observance of more or less stringent preventive measures."

From this it will be seen that the new Journal is a contribution to the domain of preventive medicine.

The present number, a large quarto, is full of tables of mortality from various diseases, all of which are separately named, and in all the States of the Union. These are followed by tables of climatology specially selected from particular Weather Bureau Stations, for purposes of comparison with the preceding tables of mortality, and last of all a series of charts or maps of the United States showing all the places which have the same mean temperature, the same mean rain-fall, and other similar data.

